# A retrospective study of imaging characteristics of mucinous tubular and spindle cell carcinoma in the kidney

**DOI:** 10.3389/fonc.2025.1515569

**Published:** 2025-03-20

**Authors:** Hong Wang, Xiaoyan Peng, Lutong Li, Yujia Yang

**Affiliations:** ^1^ Department of Medical Ultrasound, West China Hospital of Sichuan University, Chengdu, China; ^2^ West China Clinical Medical College of Sichuan University, West China Hospital of Sichuan University, Chengdu, China

**Keywords:** mucinous tubular and spindle cell carcinomas, kidney, contrast-enhanced ultrasound, contrast-enhanced computed tomography, diagnosis

## Abstract

**Purpose:**

To strengthen the recognition of mucinous tubular and spindle cell carcinomas of the kidney (MTSCC) by analyzing ultrasound and computed tomography findings.

**Materials and methods:**

This study retrospectively enrolled eleven patients with pathologically confirmed mucinous tubular and spindle cell carcinomas from 2007 to 2022. The clinical, imaging, pathological features, and prognosis of all included patients were analyzed. All imaging features were evaluated in consensus by two genitourinary radiologists.

**Results:**

All patients (48 ± 17 years, male to female, 3:8) presented with a solitary renal tumor with a mean diameter of 6.3 cm. Most of the lesions were located in the renal cortex. In ultrasonography, all 11 patients underwent conventional ultrasound and color Doppler flow imaging, and only three underwent contrast-enhanced ultrasound. In computed tomography (CT) examination, 8 of the 11 patients underwent plain CT and contrast-enhanced CT, and 1 patient underwent plain CT only. Grayscale ultrasound image demonstrated that most of the lesions were homogeneously hypoechoic with clear boundaries and regular shapes. Color Doppler flow imaging showed spotty blood flow in some cases. Contrast-enhanced ultrasound showed heterogeneous mild enhancement, and the contrast agent showed ‘slow in and simultaneous/fast out’ pattern. Plain CT showed equal or low density. CECT scanning showed slight heterogeneous enhancement in 6 patients, mild homogeneous enhancement in 2 patients. All lesions showed no hemorrhage, cystic degeneration or necrosis. Contrast-enhanced CT and contrast-enhanced ultrasound showed typical low-vascular tumors.

**Conclusion:**

MTSCC are more common in middle-aged with a significant female preponderance. CT and ultrasound showed hypovascular tumors. Preoperative imaging diagnosis is difficult. It is necessary to distinguish from other hypovascular renal tumors.multimodal imaging may be helpful for preoperative diagnosis.

## Introduction

Mucinous tubular and spindle cell carcinoma (MTSCC) of the kidney, which has been recently added to the World Health Organization Classification of Renal Tumors (1), is a rare epithelial neoplasm with low malignant potential (2). According to the 2022 World Health Organization Classification (3), MTSCC accounts for less than 1% of all renal cell carcinoma, and surgical resection is the main treatment method for MTSCC. At present, the imaging reports of MTSCC are mostly case reports or only a small number of cases are included (4–7). Owing to paucity of literature on MTSCC, any additional data would be helpful to strengthen the recognition of MTSCC. We retrospectively reviewed the clinical data of patients diagnosed with MTSCC at our institution between January 2007 and December 2022 and analyzed the clinical, imaging, pathological features, and prognosis of MTSCC, to improve diagnostic reliability. We present the following article in accordance with the STROBE (Strengthening the Reporting of Observational studies in Epidemiology).

## Materials and methods

### Patient data acquisition

The Institutional Review Board of our institution approved this retrospective study and waived the need for informed consent. We retrospectively and continuously collected the data of eleven patients with pathologically confirmed MTSCC from January 2007 to December 20222. Prior to each imaging examination oral or written consent of the patient was obtained. Potential risks and complications were been explained in detail. The clinicoradiological details and treatment details were obtained from Electronic Medical Records.

### Image acquisition

Eleven conventional ultrasound scans combined with color Doppler flow imaging (CDFI), 3 contrast-enhanced ultrasound (CEUS), 9 noncontrast computed tomography (CT) scans, and 8 contrast-enhanced CT (CECT) scans were available for review. There were no results of magnetic resonance imaging (MRI) and nuclear medicine imaging.

Ultrasound examinations were performed with a Resona7 ultrasound system (Mindray Medical International, Shenzhen, China) equipped with an SC6-1U (1–6 MHz), an iU22 ultrasound system equipped with a C5-1 (1–5 MHz) and an HDI 5000 ultrasound system equipped with a C5-2 (2–5 MHz) (Philips Medical Systems, Royal Philips, the Netherlands). Traditional B-mode ultrasound and CDFI were performed on each lesion. The imaging settings, such as gain, depth, and focus, were optimized to ensure clear visualization of the renal lesion according to the operator’s experience. The location (upper pole, middle and lower pole), boundary (clear or unclear), shape (regular or irregular), size (the longest diameter of the largest section of the lesion), echo characteristics (hypoechoic, hyperechoic, isoechoic), presence or absence of calcification, peripheral acoustic halo, color signal of blood flow and its neighboring structure relationship on ultrasound were analyzed. CEUS was performed in some cases and the videos were analyzed. In CEUS, dual-screen (on the screen are simultaneously displayed grayscale ultrasound and CEUS images) was used for real-time contrast-specific imaging at low mechanical index (e.g. the mechanical index setting was 0.078 in Resona7 and 0.06 in iU22). A dose of 1.2 mL of SonoVue (Bracco, Milan, Italy) suspension was injected through the patient’s cubital vein followed by a 5 mL saline flush. The timer was started when the contrast agent injection was completed. The target lesion and surrounding renal parenchyma were observed continuously. The mean overall examination time ranged from 3 to 5 minutes. The normal renal parenchyma was uses as a reference. The dynamic contrast enhancement patterns of the lesions were evaluated and analyzed.

CT examinations were performed with a uCT780 scanner (UNITED IMAGING, China), and Somatom Definition Flash (SIEMENS AG, Germany). For CECT, the contrast agent iohexol (300 mg/mL, dose 1.5 mL/kg) was injected.

### Statistical analysis

Statistical analysis was performed using IBM SPSS Statistics Version 25 (Armonk, NY, USA). The mean and the standard deviation (SD) were calculated for normally distributed data.

## Results

### Clinical manifestations of patients

A total of 11 lesions from 11 patients were included in the study. All patients presented with unilateral and solitary renal masses. There was a trend towards female predominance with eight women and three men, but the sample size was too small to confirm any statistical significance. The mean age was 48 years (SD ± 17 years) ranging between 23 years and 82 years. Eight patients (72.7%) were asymptomatic and incidentally observed during routine abdominal imaging for other unrelated reasons. Two patients (18.2%) presented with local symptoms of gross hematuria, and one (9.1%) presented with lumbodynia. The tumors were located in the left kidney in five cases and in the right kidney in six cases. A total of 45.5% (5/11) of the lesions were described in the upper pole, with 36.4% (4/11) and 18.2% (2/11) in the middle and lower poles, respectively. The longest tumor diameter ranged from 4 to 10 cm (mean 6.3 cm). All examiners performed routine urine examination, six cases of which were positive for occult blood, five cases were positive for proteinuria qualitatively, and three cases were positive for the both tests. Among all the patients, 10 patients did not undergo tumor marker examination. The case 4 was also found to have increased uterine volume and uterine effusion, so we hypothesized that the doctors examined this case for tumor markers: Cancer antigen (CA) 125 and CA72-4. And the results were positive. There were no significant positive results in the clinical biochemical indicators of all patients. The patient characteristics are summarized in **Table 1**.

**Table 1:** Characteristics of 11 MTSCC of kidney patients. .

Case	Sex/age at diagnosis	Clinical manifestation	Location	Diameter/cm	Routine urine examination	Tumor marker
1	F/44	asymptomatic	L/upper, Parenchymal	4.7	OBT ↑, PQ ↑	/
2	F/82	asymptomatic	R/middle, Both	6.4	OBT ↑, PQ ↑	/
3	F/43	asymptomatic	R/upper, Parenchymal	5.3	OBT ↑, PQ ↑	/
4	F/33	asymptomatic	R/upper, Both	5.8	negative	CA 125↑ CA72-4 ↑
5	M/39	asymptomatic	R/upper, Both	7.7	OBT ↑, PQ ↑	/
6	M/71	asymptomatic	L/lower , Exophytic	5	PQ ↑	/
7	F/23	gross hematuria	L/lower, Both	9.3	OBT ↑, PQ ↑	/
8	F/55	asymptomatic	L/middle, Both	4	OBT ↑	/
9	F/44	gross hematuria, lumbodynia,	R/middle, Both	11	negative	/
10	F/63	asymptomatic	R/upper, Both	2.5	OBT ↑	/
11	M/36	asymptomatic	L/middle, Exophytic	3.9	negative	/

F, female; M, male; R, right kidney; L, left kidney; OBT, occult blood test; PQ, proteinuria qualitative; ↑ indicates that the indicator is higher than the upper limit

### Ultrasonographic features

**Figure 1:**
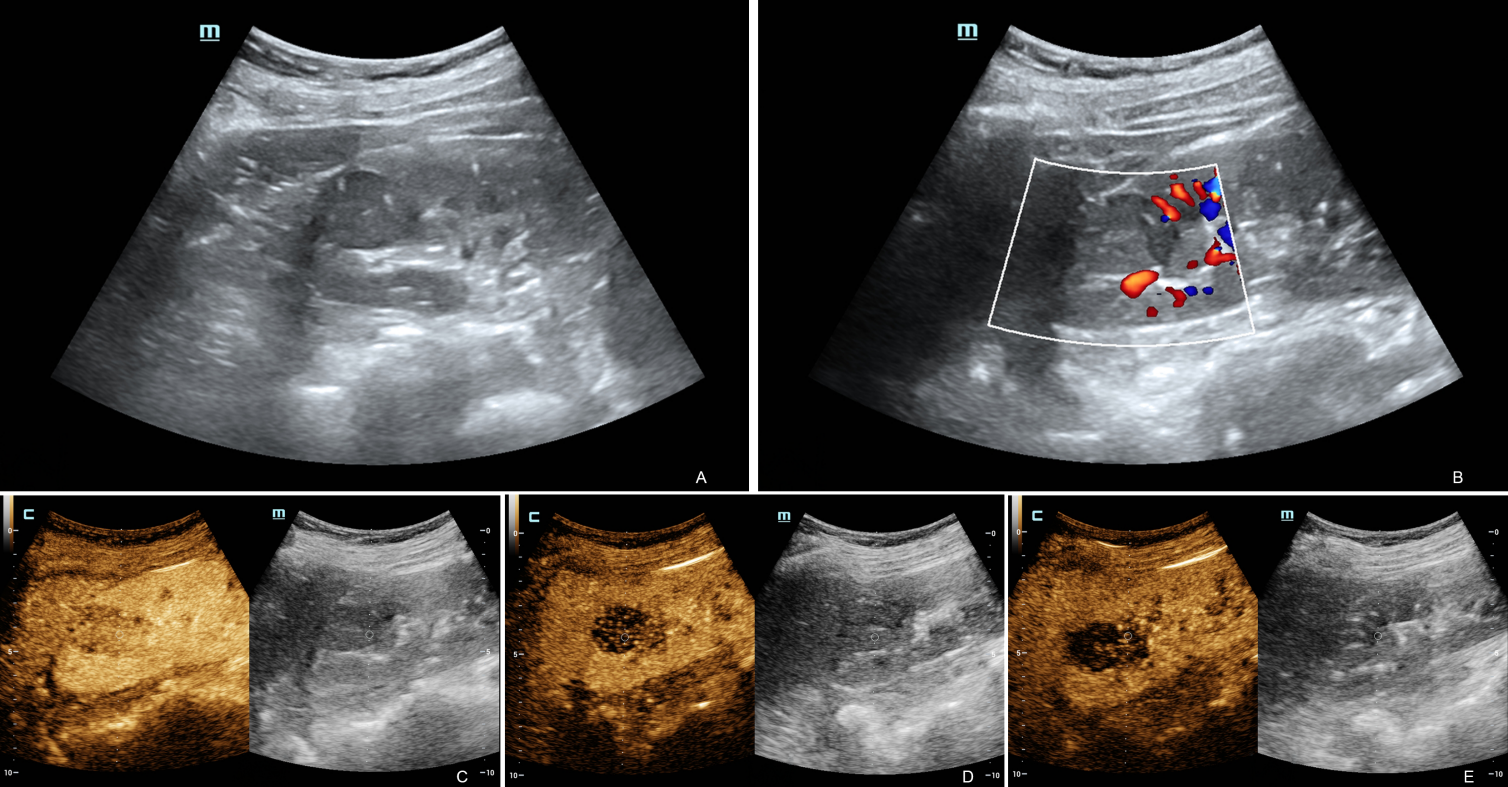
Ultrasound and contrast-enhanced ultrasound images of MTSCC. Ultrasound image showing a homogenous mass in the upper pole of the right kidney **(A)**. Color Doppler ultrasound showed no obvious blood flow in the mass, but there was some around it **(B)**. CEUS showed heterogeneous mild enhancement in the MTSCC lesion compared to the adjacent renal parenchyma in the cortical phase **(C)**. In the medullary phase **(D)**, the washout of the tumors was more simultaneous than that of the adjacent renal parenchyma. The lesion showed hypoenhancement during the delayed phase **(E)**. These performances indicated hypovascular renal tumors.

Eight patients underwent conventional abdominal ultrasound and CDFI. Three patients underwent conventional ultrasound, CDFI and CEUS. Most of the lesions (10/11) were located in the renal cortex and showed clear boundaries and regular shapes (**Figure 1A**). The only lesion left was located in the cortex-medullary junction and showed a fuzzy boundary and irregular shape on conventional ultrasound. Only one case showed calcification within the lesion. Another case displayed an acoustic halo. Using renal parenchymal echo as a reference, the grayscale ultrasound image demonstrated that most of the lesions (9/11) were homogeneously hypoechoic. One case was homogeneously isoechoic, and the only lesion left was heterogeneously hypoechoic. In CDFI, 3 cases showed internal dotted or linear blood flow signals, and 8 cases showed no internal blood flow signal (**Figure 1B**). Six cases showed peripheral dotted blood flow and there was no obvious peripheral blood flow signal around the masses in the other five cases. CEUS imaging studies were completed successfully in 3 patients with satisfactory imaging quality. In the cortical phase, three lesions showed mild enhancement. All of their enhancement was rather heterogeneous and later than that of the adjacent renal cortex. In the medullary phase, the washout of the tumors was earlier (2/3) or simultaneous (1/3) than that of the adjacent renal parenchyma. Three lesions showed hypo-enhancement during the delayed phase (**Figures 1C-E**). Tumors displayed no cystic degeneration in conventional abdominal ultrasound and CEUS. The ultrasound characteristics of the tumors are summarized in **Table 2**. Of the 11 patients who underwent routine ultrasound, 8 lesions were reported only solid renal tumors on ultrasound and 3 were suspected of renal malignancy. Of the 3 patients who underwent CEUS, two lesions were suspected to be RCC, and one only was reported malignant renal tumors without further diagnosis of the tumors.

**Table 2:** Imaging features for all tubular mucinous renal tumours with spindle cells.

Case	Ultrasound features					CT features	
	Echogenicity	Margin	Internal CDFI	Peripheral CDFI	CEUS	plain CT	CECT
1	heterogeneously hypoechoic	well-marginated	yes	yes	/	Well-circumscribed heterogeneous	/
2	homogenously hypoechoic with calcification	fuzzy boundary	no	no	heterogeneous,slow in and fast out	/	/
3	homogenously hypoechoic	well-marginated	yes	yes	/	Well-circumscribed homogenous	Heterogeneous, mild enhancement
4	homogenously hypoechoic	well-marginated	yes	no	/	Well-circumscribed homogenous	Heterogeneous, mild enhancement
5	homogenously hypoechoic	well-marginated	no	no	/	Well-circumscribed homogenous	Heterogeneous, mild enhancement
6	homogenously hypoechoic	well-marginated	no	no	/	Well-circumscribed homogenous	Heterogeneous, mild enhancement
7	homogenously hypoechoic	well-marginated	no	no	/	Well-circumscribed heterogeneous	Heterogeneous, mild enhancement
8	homogenously isoechoic	well-marginated	no	yes	/	Well-circumscribed homogenous	Heterogeneous, mild enhancement
9	homogenously hypoechoic	well-marginated	no	yes	/	/	/
10	homogenously hypoechoic with acoustic halo	well-marginated	no	yes	heterogeneous,slow in and simultaneous out	Well-circumscribed homogenous	Homogenous, mild enhancement
11	homogenously hypoechoic	well-marginated	no	yes	heterogeneous, slow in and fast out	Well-circumscribed homogenous	Homogenous, mild enhancement

CDFI, color Doppler flow imaging; CEUS, contrast-enhanced ultrasound; CT, computed tomography; CECT, contrast-enhanced computed tomography.

### CT features

**Figure 2:**
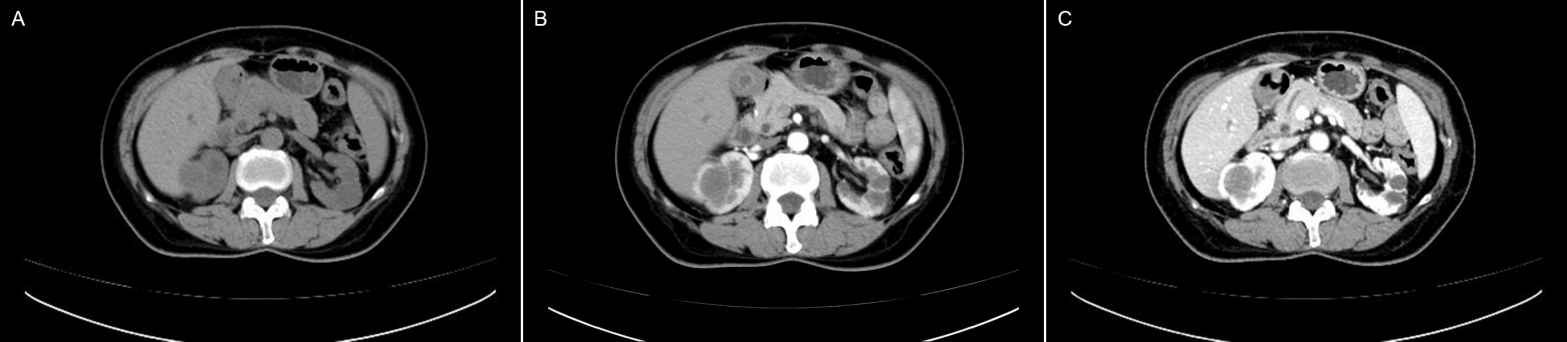
Computed tomography and contrast-enhanced computed tomography images of MTSCC: **(A)** CT plain scans showed a homogeneous slightly low density mass in the right kidney. Enhanced CT showed homogeneous mild delayed enhancement in arterial **(B)** and venous phases **(C)**.

Eight patients underwent plain CT and CECT. One patient underwent plain CT. All tumors grew expansively with a spherical or ovoid shape on CT images and had well-demarcated margins. Plain CT showed equal or low density. Compared to the normal renal parenchyma in the study, most of tumors (7/9) show homogenous in plain CT. A calcification was observed in only one patient from our series. CECT scanning showed slight heterogeneous enhancement in 6 patients, mild homogeneous enhancement in 2 patients. All lesions exhibited slow and progressive enhancement in the late phases. All lesions showed no hemorrhage, cystic degeneration or necrosis. **Figure 2** showed the plain CT and CECT performance of Case 10. All CT results (including plain CT and CECT) of 9 patients were reported malignant renal tumors without further diagnosis of the tumors.

### Histopathological features

Ten patients underwent radical nephrectomy and one patient underwent nephron-sparing surgery. Of the 11 patients, 10 underwent radical nephrectomy. Only in the case 1, due to the hydronephrosis after kidney stone surgery on the opposite kidney, nephron-sparing surgery was performed to maximize the preservation of normal renal tissue in order to protect renal function. No cancer involvement was found at the incisal margin of the submitted tissue in all cases. Gross tumor findings were available in 11 patients. These tumors are macroscopically well circumscribed and solid with a homogenous tan, gray-pink, or pale yellow cut surface. In our series, tumor size, as measured grossly in resected specimens, varied between 4 and 10 cm. Microscopically, the tumor is composed of a mixture of tubular and spindle cell components separated by variable amounts of mucinous or myxoid stroma. Immunohistochemistry (IHC) (**Table 3**) was performed in all lesions except case 8. In case 8, the diagnosis was solely given on the classic histomorphological features of the tumor without the use of any ancillary IHC technique. The positive immunohistochemical expression was as follows: RCC (6/8), PAX-8 (3/3), and PAX-2 (4/4), CK7 (10/10), Vimentin (4/4), and EMA (6/6), and AMACR (6/6), CA9 (0/4), CD117 (0/5), CD10 (2/9).

**Table 3:** Immunohistochemistry profile of cases.

Case	Vimetin	CK7	EMA	CD10	RCC	AMACR	PAX-8	PAX-2	CD117	CA9
1		+	+	−	+		+		−	−
2		+	+	−						
3		+	+	−	−				−	−
4	+	+		−	+	+		+	−	
5		+	+	+	+	+	+			
6		+	+	−	−	+	+	+		
7	+	+		+	+					
8										
9		+	+	−		+				
10	+	+			+	+		+	−	−
11	+	+		−	+	+		+	−	−

### Follow-up

All patients were alive and no metastases or recurrent evidence had been found in these patients during 41–124 months (mean 55 months) of follow-up with conventional ultrasound and/or CT once 6 months and 1 year thereafter after surgery.

Patient with localized disease treated with resection had generally favorable outcomes.

## Discussion

Histologically, the classic histomorphology of MTSCC reveals spindle cells, tubules, and mucinous stroma, delineating it from other subtypes of renal cell carcinoma (3). With the development of IHC technology, nonclassic patterns of MTSCC, including mucin-poor tumors and those showing focal papillary change have been reported recently (8). MTSCC with focal neuroendocrine differentiation or sarcomatoid change has been described (9–13). Similar to previous reports (14, 15), our report shows a female predominance (72.3%). In our study, the median age at diagnosis was 48 years (range 23-82), consistent with the literature where MTSCC occurs in adults across a broad age range (15). Patients who suffer from MTSCC are generally asymptomatic (16, 17), and indeed, only two of eleven patients presented with gross hematuria and one with lumbodynia in our study. All patients in our study presented with a solitary tumor in the kidney. Consistent with previous studies (3, 14, 17), most lesions in our study were located in the renal cortex. Radical nephrectomy or nephron-sparing surgery can be selected as the treatment method for MTSCC (18). The choice of surgical method needs to be comprehensively evaluated according to the patient’s physical condition, clinical stage of the tumor, renal function, and concomitant diseases. The 11 patients in our study survived for a long time after radical nephrectomy or nephron-sparing surgery, without local recurrence or metastasis.

Preoperative imaging diagnosis of MTSCC was difficult in our study. CEUS and CECT showed enhancement of the lesions, but the enhancement was less than the adjacent renal parenchyma, leading to the diagnosis of MTSCC needed to be distinguished from other hypovascular renal tumors, like papillary RCC and chromophobe RCC. There are few studies on the imaging characteristics of MTSCC, and most of them focus on CT and MRI findings (7, 15, 17), with only a few reports on ultrasonographic manifestations. According to literature review, it was found that Zhang Q et al. (16) reported 6 cases of conventional ultrasound, CEUS and CECT, and Ling C et al. (19) reported 7 cases of conventional ultrasound, and CECT. In our study, most of the cases were hypoechoic in conventional ultrasound, which was consistent with Ling C et al’ s study (19). In Zhang Q et al’ s study (16) study, conventional ultrasound showed that all tumors were hypoechoic. All cases in our study were solid, consistent with Zhang Q et al’ s study (16), while Ling C et al. (19) reported 1 case with solid-cystic tumor. In our study, internal blood flow signals appeared in 3 cases. Ling C et al. (19) also reported the presence of internal blood flow signals in two tumors, while in Zhang Q et al’ s study (16), ultrasound showed no internal blood flow in all cases. In our study, 6 patients were found to have peripheral point-like blood flow, and the presence of tumor peripheral blood flow signal were reported in Ling C et al’ s study (19) and Zhang Q et al’ s study (16) study. In our study, three cases of CEUS showed mild low enhancement, which was consistent with Zhang Q et al’ s study (16).

In our study, plain CT showed equal or low density and CECT showed mild low enhancement Additionally, 6 cases of CECT with heterogeneous pattern of enhancement and 2 cases with homogenous pattern of enhancement were reported in our study. Ling C et al. (19) also reported that most tumors (5/7) showed a pattern of heterogeneous enhancement. However, in Zhang Q’s study et al. (16), all cases showed homogenous pattern of enhancement. There are articles (2, 20, 21) showing that tumors less than 5 cm usually demonstrate a homogenous pattern of enhancement, whereas those larger than 5 cm are heterogeneous. The enhancement pattern may be related to the size of the tumor, However, in our study, the tumor in case 8 with a maximum diameter of less than 5cm showed a homogenous pattern of enhancement. Due to the lack of studies with large samples, the relationship between the enhancement pattern and the size of the tumor needs to be further investigated.

Currently, the organizational origin of MTSCC has not been fully elucidated. Some studies (22–24) have suggested that MTSCC originates from the epithelium of the collecting duct of the kidney, but some studies (25, 26) have now suggested that MTSCC is a low-grade malignant tumor that may originate from the distal convoluted tubules of the kidney. In our study, most MTSCC cases were RCC (6/8), PAX-8 (3/3), and PAX-2 (4/4) positive, consistent with literature reports (27). The molecular markers of distal convoluted renal tubular cells, CK7 (10/10), Vimentin (4/4), and EMA (6/6), were positive, consistent with literature reports (28, 29). However, there are also markers associated with proximal convoluted tubules that are positive, such as AMACR (6/6). These results show varying degrees of evidence of proximal and distal tubular differentiation, which suggested that this tumor has both proximal and distal renal tubular origins.

This study had several limitations. First, this study was retrospective, with a small sample size. Second, all included cases did not undergo MRI regretfully, resulting in the lack of MRI imaging features in this study. Finally, due to different types of scanners, the imaging parameters were inconsistent, which may lead to different interpretations of the results.

In conclusion, we reported eleven cases of MTSCC of the kidney. The clinical and imaging performance were described. Our research showed that MTSCC mostly occurs in middle-aged with female predominance. On imaging, lesions were often hypovascular pattern on CECT and CEUS. Preoperative imaging diagnosis was difficult. The imaging features should be validated in the future studies.

## Data Availability

The raw data supporting the conclusions of this article will be made available by the authors, without undue reservation.
